# Differences in *AMY1* Gene Copy Numbers Derived from Blood, Buccal Cells and Saliva Using Quantitative and Droplet Digital PCR Methods: Flagging the Pitfall

**DOI:** 10.1371/journal.pone.0170767

**Published:** 2017-01-26

**Authors:** Delicia Shu Qin Ooi, Verena Ming Hui Tan, Siong Gim Ong, Yiong Huak Chan, Chew Kiat Heng, Yung Seng Lee

**Affiliations:** 1 Department of Paediatrics, Yong Loo Lin School of Medicine, National University of Singapore, Singapore; 2 Division of Endocrinology and Diabetes, Khoo Teck Puat-National University Children's Medical Institute, National University Hospital, National University Health System, Singapore; 3 Singapore Institute for Clinical Sciences, A*STAR, Singapore; 4 Biostatistics Unit, Yong Loo Lin School of Medicine, National University of Singapore, Singapore; University of Hyogo, JAPAN

## Abstract

**Introduction:**

The human salivary (*AMY1*) gene, encoding salivary α-amylase, has variable copy number variants (CNVs) in the human genome. We aimed to determine if real-time quantitative polymerase chain reaction (qPCR) and the more recently available Droplet Digital PCR (ddPCR) can provide a precise quantification of the *AMY1* gene copy number in blood, buccal cells and saliva samples derived from the same individual.

**Methods:**

Seven participants were recruited and DNA was extracted from the blood, buccal cells and saliva samples provided by each participant. Taqman assay real-time qPCR and ddPCR were conducted to quantify *AMY1* gene copy numbers. Statistical analysis was carried out to determine the difference in AMY1 gene copy number between the different biological specimens and different assay methods.

**Results:**

We found significant within-individual difference (p<0.01) in *AMY1* gene copy number between different biological samples as determined by qPCR. However, there was no significant within-individual difference in *AMY1* gene copy number between different biological samples as determined by ddPCR. We also found that *AMY1* gene copy number of blood samples were comparable between qPCR and ddPCR, while there is a significant difference (p<0.01) between *AMY1* gene copy numbers measured by qPCR and ddPCR for both buccal swab and saliva samples.

**Conclusions:**

Despite buccal cells and saliva samples being possible sources of DNA, it is pertinent that ddPCR or a single biological sample, preferably blood sample, be used for determining highly polymorphic gene copy numbers like *AMY1*, due to the large within-individual variability between different biological samples if real time qPCR is employed.

## Introduction

There has been recent interest in copy number variants (CNVs) of the human salivary amylase (*AMY1*) gene and its association with obesity via carbohydrate metabolism [1]. *AMY1* gene, encoding salivary α-amylase is located in a gene cluster on 1p21. Evolutionary analysis revealed that *AMY1* gene was derived from duplication of an ancestral pancreatic amylase gene, with the insertion of a retrovirus sequence that is responsible for tissue-specific expression [2]. The *AMY1* gene has a very high sequence identity showing extensive CNV ranging from 2 diploid copies (1 paternal and 1 maternal) to as many as 16 copies [3]. The CNV of *AMY1* is strongly correlated with the amount of α-amylase in saliva [3–5]. Low levels of *AMY1* CNVs were associated with decreased levels of α-amylase in the saliva and an increased risk of obesity [1], while higher AMY1 copy number was associated with a protective effect against obesity in Mexican children [6]. In addition, females with early-onset obesity were shown to have lower AMY1 copy number and the AMY1 copy number was found to have a significant inverse correlation with body fat percentage and BMI [7]. However, a study by Usher CL et al that examined amylase gene copy number in a cohort of ~3500 obese and lean individuals found no significant association between AMY1 copy number and BMI [8]. It can be postulated that the lack of association may be attributed to the different methodologies employed in assessing AMY1 gene copy number in the samples, as Usher CL et al used ddPCR and computational analysis of whole genome sequencing data [8] which were postulated to have higher precision over qPCR technique used in previous reports which showed correlation between AMY1 copy number and obesity [1].

Until recently, blood samples are the preferred choice of genetic material because they yield sufficient amount of genomic DNA for genetic studies [9]. The quality of genomic DNA from blood is high without contamination of foreign DNA [10]. However, the collection of blood samples may not always be possible. Study subjects may be reluctant to provide blood samples, thereby reducing participating rates. Therefore, less invasive procedures for collecting DNA samples are prevalent.

Several studies have found that exfoliated buccal epithelial cells are promising alternative sources of DNA [11–14]. Another alternative source of DNA is whole saliva which is reported to yield DNA with better quality than from buccal swabs [15]. DNA extracted from saliva was reported to have a DNA methylation pattern more similar to that of brain tissues as compared to DNA extracted from blood [16]. Recently, a study also showed that saliva collected using the Oragene kit yielded high quantity and high quality DNA for genetic studies [17]. However, there are still concerns over using DNA extracted from saliva samples or buccal cyto-brushes for genotyping [18, 19] due to possible bacterial contamination which may affect yield and quality of DNA [20]. Furthermore, there is lack of data on whether different biological samples can be used interchangeably for the genotyping of a specific gene of interest, especially for complex genes like the *AMY1* gene.

The performance of different analytic methods using DNA from buccal cells, saliva and blood for the determination of AMY1 gene copy number is currently unknown. To our knowledge, no study to date has evaluated whether there are within-individual differences in *AMY1* gene copy number between DNA derived from blood, buccal cells or saliva. It is logical to assume concordance in gene copy numbers as they are derived from the same person. However, DNA collected using different methods were shown to differ in quality and quantity [18]. Furthermore, differences in copy number variants are observed in different tissues of the same individual [21]. Our original research aim was to determine if *AMY1* CNV quantified by TaqMan quantitative polymerase chain reaction (qPCR) is comparable between DNA samples derived from blood, buccal and salivary samples. However, we found unexplained variability and hypothesized that these apparent within-individual differences in *AMY1* gene copy number in DNA samples derived from blood, cheek cells and saliva may either be attributed to differences in DNA quality or limitation of the quantitation method. Hence, in this study, we compared the *AMY1* gene copy number in DNA derived from blood, cheek cells and saliva of the same individual using qPCR and an alternative quantitation method, droplet digital PCR (ddPCR).

## Methods

### Participants

Seven male participants were recruited for this study. These participants were part of a research study investigating ethnic differences in glycemic and insulin responses [22, 23]. All participants underwent a screening visit to assess eligibility, which comprised of a health assessment, anthropometric measurements and a health questionnaire (relating to medical history, smoking habits, history of any illness and use of any medications). Those who fulfilled all acceptable criteria (BMI 18.5 to <25kg/m^2^; age 18–45 years; blood pressure 110–120 / 70–90 mmHg; fasting blood glucose 4–6 mmol/L; not on prescription medication; non-smoking; no genetic or metabolic diseases) were included in the study. This study was approved by the National Healthcare Group Domain Specific Review Board, Singapore. The study was registered at clinicaltrials.gov as NCT01804738 and all participants provided written informed consent prior to their participation in the study.

### Blood collection

After an overnight fast, 300μL of capillary blood (obtained from finger pricks) was collected into Microvette® capillary blood collection tubes treated with di Potassium EDTA (CB 300 K2E, Sarstedt, Germany) and kept on ice immediately. The blood samples were transferred and stored at −80°C until DNA extraction [23].

### Buccal cells

For the collection of buccal cells, participants were asked to thoroughly rinse out their mouth twice with water. Buccal cells were collected using sterile foam-tip buccal cell collection swabs (Catch-All^TM^ Sample Collection Swab, Epicentre, Madison, WI, USA) by rolling the collection swab firmly on the inside of the cheek for approximately 20 times on each side, making certain to move the swab over the entire cheek. The swab samples were air dried for 30 minutes at room temperature and then stored in original packaging at -80°C until DNA extraction.

### Saliva

In preparation for saliva collection, participants were instructed to empty their mouths by swallowing all saliva and to rinse their mouth thoroughly with water in order to reduce bacteria, fungi and potential food remnants [9]. Whole saliva was collected using Oragene^®^ DNA self-collection kit (DNA Genotek, Ottawa, ON, Canada). Participants were asked to deposit approximately 2 ml saliva through the funnel into the collection tube until the amount of liquid saliva (excluding bubbles) reaches the fill line. When an adequate sample was collected, the funnel lid was then closed firmly. The collection tube was designed so that stabilizing liquid that is attached to the lid mixes with the saliva when the funnel cap is securely fastened. This starts the initial phase of DNA isolation and stabilizes the saliva sample for long-term storage [24]. Saliva samples were stored at 4°C until DNA extraction.

### DNA extraction

DNA was extracted from whole blood using QIAamp^®^ DNA Mini Kit (Qiagen, Netherlands) as described by the manufacturer. For buccal cells, DNA was also extracted using QIAamp^®^ DNA Mini Kit (Qiagen, Netherlands), modified for the extraction of DNA from buccal swabs. DNA was extracted from the saliva samples using the Oragene kit (DNA Genotek, Ottawa, ON, Canada) as described by the manufacturer. All extracted DNA samples were quantified and stored at -20°C until analysis.

### Quantification of DNA

The concentration of DNA sample was measured using the absorbance method (NanoDrop 1000 v3.7.1, Thermo Scientific). Absorbance of ultraviolet light at wavelengths of 260 nm and 280 nm were used to calculate the optical density (OD) 260/280 ratio. These values are used to compare the ratio of nucleic acid concentration in the sample (OD 260 nm) to that of protein and organic contaminants (OD 280 nm). A ratio of 1.8 was considered ideal for the OD 260/280 ratio, indicating minimal protein and organic contamination. The average DNA concentration was taken from 2 readings. All DNA samples were diluted with nuclease free water and the concentration was verified by Nanodrop.

### Quantitative polymerase chain reaction (qPCR) for the *AMY1* gene

The qPCR method used to determine diploid *AMY1* gene copy numbers was adapted from previously published work [4]. The primers and probes of the TaqMan qPCR assay employed in our analyses specifically target a region within exon 1 of the human *AMY1* gene, which is absent in the *AMYP1* pseudogene, therefore ensuring specificity of the qPCR assay for *AMY1*. Briefly, each reaction consisted of 10ul of TaqMan Genotyping Master Mix (Applied Biosystems, Foster City, CA), 1ul of TaqMan Copy Number Assay specific for human *AMY1* (Hs07226362-FAM), 1ul of RNAse P reference assay (44003328-VIC), 4ul of DNA (5ng/ul) and 4 ul of nuclease free water. The samples were run in quadruplicates on ABI Prism 7500 Real-Time PCR System (Applied Biosystems). Each individual’s blood, buccal cells and saliva DNA were present in the same plate. The study samples were analyzed using Copy Caller Software v2.0 (Applied Biosystems). *AMY1* diploid copy number was estimated using a standard curve constructed from a reference DNA sample NA18972 (Coriell Cell Repositories, Camden, NJ) previously determined to have 14 diploid copies of AMY1 by qPCR and Fiber FISH [3, 25].

### Droplet digital PCR (ddPCR) for *AMY1* gene copy number analysis

Copy number of the *AMY1* gene was determined using the QX200 Droplet Digital PCR (ddPCR) System (Bio-Rad Laboratories, Hercules, CA) according to the manufacturer's instructions. Briefly, each reaction consisted of 11μl ddPCR Supermix for probes (no UTP), 1μl of human *AMY1* assay (dHsaCP1000594: 900nM primers and 250nM FAM probe), 1μl of AP3B1 assay (dHsaCP1000001: 900nM primers and 250nM HEX probe), 1μl HaeIII restriction enzyme (3U/μl), 6μl of nuclease free water, and 2μl of DNA (10ng/μl). 20μl of each reaction was transferred to a droplet generation cartridge and 70μl of Droplet Generation Oil was added. Droplets generated with QX200 Droplet Generator (~40ul) were loaded into a clean 96-well PCR plate and the plate was sealed with foil seal using PX1 plate sealer. PCR amplification was performed in a C1000 Touch Deep Well PCR System with the following conditions: 10 minutes at 95°C, 40 cycles of 94°C for 30 seconds and 60°C for 60 seconds with ramp rate of ~2°C/second followed by 98°C for 10minutes with ramp rate of ~1°C/second. Droplets were read in a QX200 Droplet Reader and analysis was performed using QuantaSoft v1.6.6.0320 (Bio-Rad Laboratories). A negative control (water) and a sample known to have 14 diploid copies of AMY1 gene (NA18972, Coriell Cell Repositories, Canden, NJ) were included in the run. Study samples were analyzed in triplicates.

### Statistical analysis

Data was presented as mean ± standard deviation (SD). We performed ANOVA to compare the difference in *AMY1* gene copy number between blood, buccal swab and saliva samples from each subject. Post-hoc bonferroni adjustment was applied for ANOVA analysis. Paired Student’s t-test was used to determine the difference in *AMY1* gene copy number between qPCR and ddPCR in blood, buccal swab and saliva respectively. A *p*-value of <0.05 was considered statistically significant. Statistical analyses were conducted using SPSS version 23.0 (SPSS Inc., Chicago, IL, USA).

## Results

### Quantification of DNA extracted from blood, buccal swab and saliva

The quantity and purity of DNA measured by absorbance method (NanoDrop) are shown in **Table 1**. The estimated amount of DNA extracted from 200 μl blood samples varied between 45.7 to 150.3ng/μl with a mean of 72.7 ng/μl, from 1 buccal swab between 20.1 to 83.6 ng/μl with a mean of 48.3 ng/μl, and from 500 μl Oragene saliva samples between 50.0 to 453.5 ng/μl with a mean of 227.2 ng/μl. The quality of DNA obtained was assessed by the ratio of absorbance at 260 nm and 280 nm. The mean values of the optical density 260/280 ratios were 1.90, 1.92 and 1.89 for genomic DNA purified from blood, buccal swab and saliva respectively.

**Table 1:** Comparison of DNA yield and quality according to DNA collection method.

**Method of DNA collection**	**Blood**		**Buccal cells (swabs)**		**Saliva**	
**Amount of sample used**	200 μl	*Range*	1 swab	*Range*	500 μl	*Range*
**DNA concentration (ng/ μl)**	72.7 ± 35.5	45.7–150.3	48.3 ± 20.1	20.1–83.6	227.2 ± 159.0	50.0–453.5
**Optical density 260/280 nm ratio**	1.90 ± 0.05	1.83–1.96	1.92 ± 0.06	1.84–2.00	1.89 ± 0.05	1.80–1.94

Data are presented as mean ± SD

### Determination of *AMY1* gene copy number by qPCR

**Fig 1:**
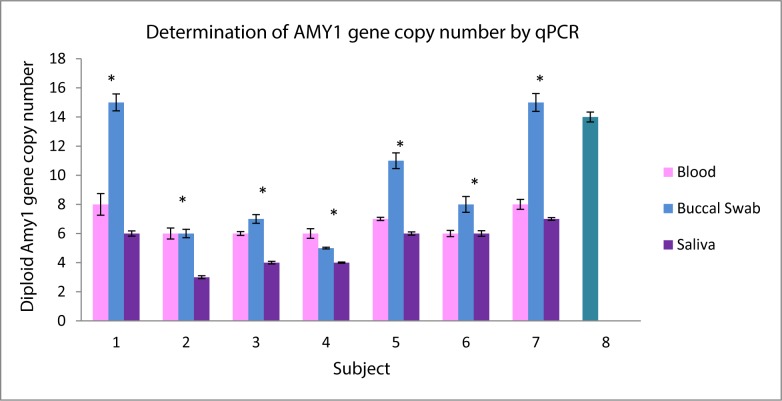
Determination of *AMY1* gene copy number by qPCR. 1–7 represents each of the 7 subjects recruited for this study, 8 represents the positive control known to have 14 diploid copies of *AMY1* gene. The bars represent the different DNA samples extracted from blood, buccal swab and saliva of each individual. * denotes significant difference of p<0.01 (after bonferroni adjustment) between the different biological samples in each subject using ANOVA analysis.

ANOVA analysis was used to compare the difference in *AMY1* gene copy number between DNA samples extracted from blood, buccal swab and saliva of each subject. By the qPCR method, the average *AMY1* gene copy number in the 7 subjects were 7 (range: 6–8) in blood samples, 10 (range: 5–15) in buccal swab samples and 5 (range: 3–7) in saliva samples. In each of the 7 subjects whose DNA samples were analyzed using qPCR, there was at least one significant difference between the different biological samples (either blood vs. buccal swab, blood vs. saliva or buccal swab vs. saliva) with p-value<0.01 after bonferroni adjustment **(Fig 1)**.

### Determination of *AMY1* gene copy number by ddPCR

**Fig 2:**
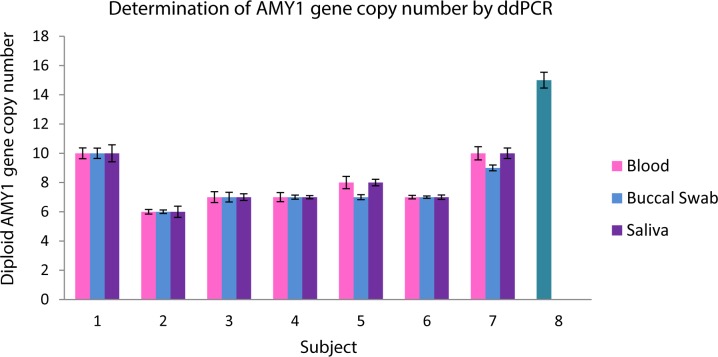
Determination of *AMY1* gene copy number by ddPCR. 1–7 represents each of the 7 subjects recruited for this study, 8 represents the positive control known to have 14 diploid copies of *AMY1* gene. The bars represent the different DNA samples extracted from blood, buccal swab and saliva of each individual.

The *AMY1* gene copy number as determined by ddPCR is comparable between the different biological specimens in each subject. The average *AMY1* gene copy number in the 7 subjects were 8 (range: 6–10) in blood samples, 8 (6–10) in buccal swab samples and 8 (range: 6–10) in saliva samples. Although subject 5 and 7 demonstrated one copy less in their buccal swab samples compared to their blood and salivary samples, there was no significant difference (p>0.05) in *AMY1* gene copy number between the different biological samples obtained from the same subject when analyzed by ddPCR **(Fig 2)**.

### Comparison of *AMY1* gene copy number between qPCR and ddPCR

**Fig 3:**
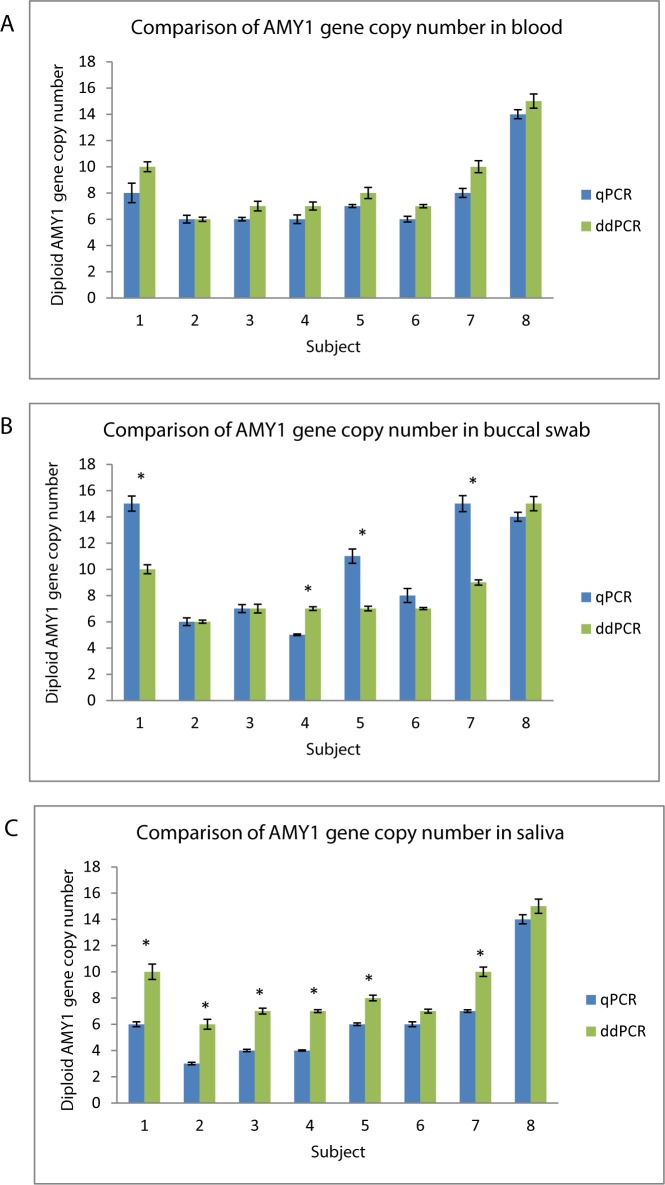
Comparison of *AMY1* gene copy number determined by qPCR and ddPCR in different biological samples. 1–7 represents each of the 7 subjects recruited for this study, 8 represents the positive control known to have 14 diploid copy number of *AMY1* gene. The bars represent the 2 different methods used to determine *AMY1* gene copy number in the samples (qPCR and ddPCR). A: Comparison of *AMY1* gene copy number in blood. B: Comparison of *AMY1* gene copy number in buccal swab. C: Comparison of *AMY1* gene copy number in saliva. * denotes significant difference of p<0.01 between qPCR and ddPCR for each type of biological sample after paired Student’s t test analysis.

We compared *AMY1* gene copy number between qPCR and ddPCR in each type of biological sample using paired Student t-test analysis. The average *AMY1* gene copy number in the blood samples were 7 (range: 6–8) as determined by qPCR and 8 (range: 6–10) as determined by ddPCR. Although AMY1 gene copy number in the blood DNA as determined by ddPCR is generally 1–2 copies higher as compared to qPCR, there was no statistically significant difference in *AMY1* gene copy number for blood DNA samples analyzed by qPCR and ddPCR in each of the 7 subjects using paired Student’s t test as shown in **Fig 3A**.

The average *AMY1* gene copy number in the buccal swab samples were 10 (range: 5–15) as determined by qPCR and 8 (range: 6–10) as determined by ddPCR. * denotes there was a significant difference (p<0.01) in the *AMY1* gene copy number for buccal swab samples determined by qPCR and ddPCR in 4 out of 7 subjects **(Fig 3B)**.

The average *AMY1* gene copy number in the buccal swab samples were 5 (range: 3–7) as determined by qPCR and 8 (range: 6–10) as determined by ddPCR. There was a significant difference (p<0.01) in the *AMY1* gene copy number for buccal swab samples determined by qPCR and ddPCR in 6 out of 7 subjects as denoted by * **(Fig 3C)**.

## Discussion and Conclusions

Genetic studies often require the recruitment of large number of participants and ascertainment of DNA samples. Hence, collection methods that do not unduly burden study participants are integral to improving participating rates for genetic epidemiologic studies. In addition to the common method of extracting DNA from blood samples obtained through venipuncture, other non-invasive sampling methods like extracting DNA from saliva and buccal cells have been introduced and used in other studies [13, 16, 26]. In this study, we were able to obtain high quality DNA of adequate yield for genotyping from the three different biological samples namely blood, buccal cells and saliva. We found the mean DNA yield from saliva to be the highest, followed by blood then buccal cells (**Table 1**). This may be attributed to the use of 500ul of saliva as compared to only 200ul of blood and a single buccal swab for DNA extraction. Nonetheless, our results showed that blood, buccal cell and saliva samples yielded good quality DNA for downstream qPCR applications as their OD260/280 values were close to the ideal value of 1.8, indicating minimal protein and organic contamination [26].

Our results showed significant within-individual variability in *AMY1* gene copy number between different biological samples as determined by real-time qPCR (**Fig 1**). This has implications on studies using real-time qPCR and different biological samples for genotyping copy number of highly polymorphic genes such as *AMY1* which is reported to have CNV ranging from 2 to 16 copies [1]. In contrast, there was no significant within-individual variability in *AMY1* gene copy number between the different biological samples as determined by ddPCR (**Fig 2).** This suggested that ddPCR which was previously shown to be a more sensitive method in the absolute quantification of gene copy number with a lower level of uncertainty (<5%) [27, 28], may be a better method for analyzing highly polymorphic genes such as AMY1 in different biological samples obtained from the same individual.

In addition, we compared the AMY1 gene copy number in each biological sample (blood, buccal swab and saliva) between qPCR and ddPCR. Although the AMY1 gene copy number in blood samples showed a consistent 1–2 copies over-read between qPCR and ddPCR, *AMY1* gene copy number in blood showed no statistically significant difference between qPCR and ddPCR **(Fig 3A)**. However, there was a significant difference in AMY1 gene copy number in both buccal swab **(Fig 3B)** and saliva samples **(Fig 3C)** between qPCR and ddPCR. The inconsistent data between the two quantification methods demonstrated that DNA from buccal cells and saliva may not be good alternatives to blood when genotyping for genes with highly variable CNVs. Previous studies showed that buccal swabs and saliva contained only 11% [13] and 68% [29] human DNA due to bacterial contamination [20]. Hence, the decreased concentration of human DNA in buccal swab and saliva samples are likely to have affected the amplification efficiency in qPCR [30]. The event counts which may be an indirect measure of DNA quantity in ddPCR also differed between the different sample types but did not significantly affect the consistency of *AMY1* gene copy number detection **(S1 Table)**.

We recognized several limitations for this study. The concentration and quality of extracted DNA was measured using NanoDrop spectrophotometry which is a less sensitive quantification method as it is not able to detect DNA fragmentation [31]. However, we ran a 1% gel electrophoresis to check for DNA degradation in samples extracted from blood, saliva and buccal swab of all 7 subjects as shown in **S1 Fig.** A distinct band with minimal DNA degradation was observed in all blood and saliva samples. DNA from buccal swab samples showed a greater degree of degradation and only a faint band was observed due to low DNA concentration. Although the presence of bacterial contamination will reduce the actual concentration of human DNA used in qPCR and may contribute to the difference in AMY1 copy number between the different biological samples, we did not measure the amount of bacterial DNA due to limited amount of samples [20]. The event counts in ddPCR and gel electrophoresis indicated that there is a difference in DNA quality and quantity between the sample types. This difference did not significantly affect the ddPCR detection of AMY1 gene copy number between the sample types but it may have affected amplification efficiency in qPCR, hence causing the significant difference in AMY1 gene copy number detected by qPCR. Therefore, we have showed that ddPCR is a more sensitive method that is independent of bacterial contamination and is able to detect consistent AMY1 copy number across different biological samples. In addition, we analyzed AMY1 gene copy number in diluted DNA samples (20ng, 40ng, 80ng) extracted from blood and saliva of one subject using qPCR. The blood DNA showed a fairly consistent AMY1 gene copy number across the diluted samples but we were only able to determine AMY1 gene copy number from the saliva DNA at a higher concentration of 80ng and the detected gene copy number was comparable to that as determined in 80ng of blood DNA **(S2 Fig)**. On contrary, diluted DNA (10ng, 20ng) from saliva, buccal swab and blood samples of the same subject showed no significant difference in AMY1 gene copy number when analyzed using ddPCR **(S3 Fig)**. These results demonstrated that qPCR analysis of AMY1 gene copy number is affected by concentration of target DNA. However, a larger sample size and inclusion of more serial dilution of samples will be required to validate these preliminary data. Another limitation of our study is the small sample size (n = 7) which did not permit us to examine ethnic and age differences that were suggested to affect the yield of DNA collection methods [13, 32]. Though we were able to detect a significant difference in *AMY1* gene copy number between the different biological specimens and different quantification methods within our small sample size, a larger sample will enable us to validate our findings.

In conclusion, our findings suggested that the determination of AMY1 gene copy number is dependent on the type of biological sample and the sensitivity of the type of assay used for copy number analysis. Despite buccal cells and saliva samples being good alternatives to blood samples to obtain high quality genomic DNA, it is pertinent that a single biological sample be used for determining *AMY1* gene copy numbers due to the large within-individual variability between different biological samples especially when using qPCR. Our data showed that DNA extracted from blood is the preferred biological sample for copy number analysis and ddPCR appeared to be more precise than qPCR method in determining AMY1 copy number with less variability across different biological samples from the same individual. This should also be applicable in the determination of other genes with highly variable copy numbers.

## Supporting Information

S1 Fig Agarose gel (1%) electrophoresis of subjects’ DNA samples. The degree of DNA degradation in the samples was analyzed by gel electrophoresis. Approximately 500ng of each DNA sample extracted from blood, buccal swab and saliva of all 7 subjects were ran at 70V on a 1% agarose gel. *denotes DNA extracted from blood, # denotes DNA extracted from buccal swab, + denotes DNA extracted from saliva and B denotes a missing buccal swab DNA sample from one of the subjects.
(EPS)

S2 Fig Comparison of AMY1 gene copy number in blood and saliva samples using qPCR. We carried out serial dilution on DNA extracted from blood and saliva samples of one subject. Taqman qPCR as described in the methods section of the manuscript was performed to analyze the AMY1 gene copy number in 20ng, 40ng and 80ng of each type of DNA sample.
(EPS)

S3 Fig Comparison of AMY1 gene copy number in blood, buccal swab and saliva samples using ddPCR. We carried out serial dilution on DNA extracted from blood, buccal swab and saliva samples of one subject. Droplet digital PCR (ddPCR) as described in the methods section of the manuscript was performed to analyze the AMY1 gene copy number in 10ng and 20ng of each type of DNA sample.
(EPS)

S1 Table Event counts for different biological samples used in ddPCR. The table shows the average accepted droplets (average event counts) in the blood DNA, saliva DNA and buccal DNA of the 7 subjects as analyzed by ddPCR (described in methods section of the manuscript). The average accepted droplets consist of average positive and negative AMY1 droplets where positive droplets are droplets above threshold level and negative droplets are droplets below threshold level. AP3B1 is consistently present at 2 copies in the genome so it was chosen as a reference assay for normalization. Although the average accepted droplets differed between blood DNA, saliva DNA and buccal DNA, the average ratio of AMY1 to AP3B1 copies per 20ul well was fairly consistent in all 3 types of samples.
(EPS)

## Abbreviations

AMY1: Human salivary amylase
qPCR: Quantitative polymerase chain reaction
ddPCR: Droplet digital polymerase chain reaction
CNV: Copy number variant
OD: Optical density
SD: Standard deviation
